# Lessons Learned by Community Stakeholders in the Massachusetts Childhood Obesity Research Demonstration (MA-CORD) Project, 2013–2014

**DOI:** 10.5888/pcd14.160273

**Published:** 2017-01-26

**Authors:** Claudia Ganter, Alyssa Aftosmes-Tobio, Emmeline Chuang, Jo-Ann Kwass, Thomas Land, Kirsten K. Davison, MA-CORD Study Group

**Affiliations:** 1 Harvard T.H. Chan School of Public Health, Boston, Massachusetts; 2 Technical University Berlin, Berlin, Germany; 3 University of California, Los Angeles, Fielding School of Public Health, Los Angeles, California; 4 Massachusetts Department of Public Health, Boston, Massachusetts.

## Abstract

**Introduction:**

Childhood obesity is a multifaceted disease that requires sustainable, multidimensional approaches that support change at the individual, community, and systems levels. The Massachusetts Childhood Obesity Research Demonstration project addressed this need by using clinical and public health evidence-based methods to prevent childhood obesity. To date, little information is known about successes and lessons learned from implementing such large-scale interventions. To address this gap, we examined perspectives of community stakeholders from various sectors on successes achieved and lessons learned during the implementation process.

**Methods:**

We conducted 39 semistructured interviews with key stakeholders from 6 community sectors in 2 low-income communities from November 2013 through April 2014, during project implementation. Interviews were audio-recorded, transcribed, and analyzed by using the constant comparative method. Data were analyzed by using QSR NVivo 10.

**Results:**

Successes included increased parental involvement in children’s health and education, increased connections within participating organizations and within the broader community, changes in organizational policies and environments to better support healthy living, and improvements in health behaviors in children, parents, and stakeholders. Lessons learned included the importance of obtaining administrative and leadership support, involving key stakeholders early in the program planning process, creating buffers that allow for unexpected changes, and establishing opportunities for regular communication within and across sectors.

**Conclusion:**

Study findings indicate that multidisciplinary approaches support health behavior change and provide insight into key issues to consider in developing and implementing such approaches in low-income communities.

## Introduction

In the United States, the prevalence of childhood obesity is high: 16.9% of children and adolescents aged 2 to 19 years were obese in 2011–2012 (1). Racial/ethnic and socioeconomic disparities between children of normal weight and obese children also persist (2–4). Obesity is a multifaceted disease, demanding sustainable, multidimensional approaches that support change at the individual, community, and systems levels (5–7). Multidisciplinary approaches are more successful in addressing childhood obesity than are single-site interventions (8,9). A 2016 review showed the promising results of multicomponent community-based interventions designed to prevent childhood obesity (10). In public health research, multidisciplinary interventions play an important role and should be emphasized (11–14). Funded by the Centers for Disease Control and Prevention, the Childhood Obesity Research Demonstration (CORD) project addressed this demand by incorporating evidence-based approaches (15). CORD is a multisite program that was implemented from September 2012 through August 2014 in Massachusetts, California, and Texas. Obesity is most prevalent in families with low socioeconomic status (4); therefore, CORD targeted underserved children aged 2 to 12 years (15).

This study focused on the Massachusetts site of CORD (MA-CORD). Evidence-based interventions were implemented in 5 community sectors: health care; early care and education; the Special Supplemental Nutrition Program for Women, Infants, and Children (WIC); schools; and after-school programs (16,17). Interventions targeted 5 key behaviors: fruit and vegetable consumption, sugar-sweetened beverage consumption, physical activity, screen time, and sleep duration. These behaviors have strong associations with children’s weight development (17). To date, little information is known about the successes and lessons learned from a stakeholder’s perspective for implementing multidisciplinary interventions. A Cochrane review called for more qualitative research as part of intervention implementation (18). Although researchers can gain valuable insight from stakeholders’ experiences with interventions such as MA-CORD, few studies provide a detailed qualitative account of the implementation process (9,18,19). This qualitative study addressed this gap by outlining successes and lessons learned from the perspective of community stakeholders directly engaged with MA-CORD, including stakeholders from after-school programs, elementary and middle schools, health care, WIC, the parks and recreation department, and coordinators from each community.

## Methods

MA-CORD was implemented in 2 communities in Massachusetts (population, 40,545 and 94,958) from September 2012 through August 2014. Poverty rates in both communities are approximately twice as high as the state’s average, with a mean income per capita between $12,600 and $14,500 lower than the state average (20,21). Both communities have large non-Hispanic white (~68%) and Hispanic (16%–22%) populations. Interventions were implemented in multiple community sectors (Figure). Details on the intervention components and evaluation design for MA-CORD are available elsewhere (16,17,22).

**Figure:**
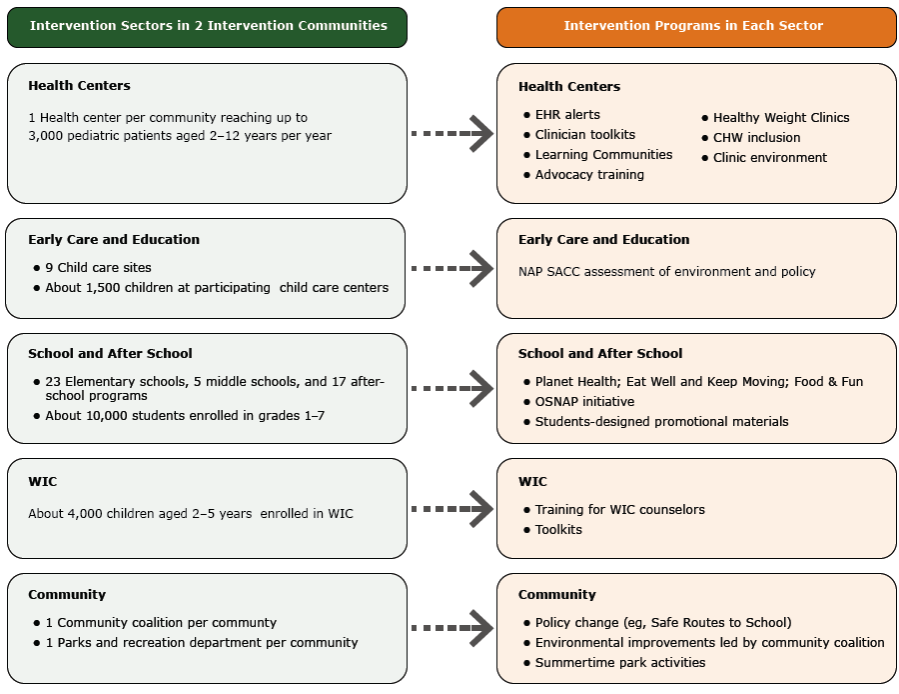
Summary of intervention sectors and intervention programs (17), study of success stories and lessons learned in Massachusetts Childhood Obesity Research Demonstration project, 2013–2014. Abbreviations: CHW, community health worker; EHR, electronic health record; NAP SACC, Nutrition and Physical Activity Self-Assessment for Child Care; OSNAP, Out-of-School Nutrition and Physical Activity; WIC, Special Supplemental Nutrition Program for Women, Infants, and Children.

Stakeholders from all sectors who were directly (eg, teachers, pediatricians) or indirectly (eg, school principals, program directors) engaged in implementing MA-CORD were invited by email from October 2013 through April 2014 to participate in an interview. We had no other inclusion or exclusion criteria. Up to 2 follow-up emails were sent; stakeholders who did not reply after the third email were counted as nonresponders. We contacted 183 stakeholders and 40 (22% response rate) completed an interview. The study was approved by the institutional review board at the Harvard T.H. Chan School of Public Health. Stakeholders received a $20 gift card as compensation.

A semistructured interview guide was developed to support standardization of interview procedure (Box). Two authors (A.A., C.G.) conducted all interviews by telephone from November 2013 through April 2014. One interview was conducted with 2 stakeholders, the previous and current coordinator from 1 community, resulting in 39 interviews with 40 participants. Demographic information was collected at the end of each interview. The average interview length was 34 minutes, with a range of 16 to 87 minutes.

**Box. Sample questions from the semistructured interview guide used for qualitative study of MA-CORD (Massachusetts Childhood Obesity Research Demonstration) project**

**Organizational and individual role in MA-CORD**

What are your organization’s and your own role in MA-CORD?

What specific things have you done as part of MA-CORD?

**Institutional fit**

Does MA-CORD fit with your organization’s priorities?

Do you feel it is a high, medium, or low priority for your organization?

What gives you that impression?

Were any competing priorities voiced by staff?

Does MA-CORD fit with your current work tasks and job description?

Can you please explain that a little bit?

Do you feel that your role and work in MA-CORD is valued and recognized?

**Successes and barriers, time commitment**

Thinking back on your experiences with MA-CORD over the past year, what do you think has been working well?

What problems or challenges (if any) have you, or the staff implementing MA-CORD, experienced?

**Parent involvement**

How, if at all, has parents’ awareness of and/or involvement in childhood obesity changed since MA-CORD was launched?

What do you think is necessary to increase parent involvement and awareness of childhood obesity prevention?

**Changes over time**

Have there been any major changes in your organization since MA-CORD started?

**Linkage**

Have you noticed any connection between MA-CORD activities within your organization and obesity prevention efforts within the broader community?

To your knowledge, have children who are overweight or obese been referred to other obesity prevention programs in your community (eg, Healthy Weight Clinic, after-school programs)?

As part of MA-CORD, do you interact with other sectors (eg, school system, health clinics, after school, child care, parks and recreation) in the community?

**Closing**

If you were giving the choice to be a part of MA-CORD again, would you choose to?

If yes: Why?

If no: Why not?

Is there anything I haven’t asked about MA-CORD that you think is important for me to know?

### Data analysis

Audio files were transcribed and transcripts were reviewed for accuracy by 1 interviewer (C.G.). Final transcripts were entered into QSR NVivo 10.0 (QSR International Pty Ltd). Data analyses were conducted by using the constant comparative method based in grounded theory. An inductive approach was used (11,23). One coder (C.G.) read 5 randomly selected transcripts representing different sectors to develop a coding framework that reflected successes and lessons learned. This framework was then discussed (A.A., C.G., K.K.D.), and 2 coders (A.A., C.G.) coded 5 additional, randomly chosen interviews. Coding was compared and discrepancies were resolved by the 2 coders (A.A., C.G.). Additional categories were also discussed and added as needed. Remaining transcripts were coded by 1 coder (C.G.). The framework was scrutinized for overlap and subcategory relevance, and a final framework (Table 1) was developed by 3 authors (A.A., C.G., K.K.D.) To attain reliability within the coding process, each decision on changes to the codebook was discussed and documented. Additional coding was conducted if needed. During data collection, an audit trail was used to track interview participants and procedures (24). All 3 coders have a background in public health and experience in qualitative research.

**Table 1:** Coding Framework, Including Main Themes, Subthemes, and Definitions for Study on Success Stories and Lessons Learned by Stakeholders (N = 40) in the MA-CORD Project, Massachusetts, 2013–2014

Main theme	Subtheme	Definition
**Success stories**	Intervention acceptability	Stakeholder’s support of MA-CORD. Includes information about whether MA-CORD was prioritized and about the organizational fit.
	Increase in parent involvement	Increase of parent participation and interest in activities related to childhood obesity (eg, participation in school programs, greater interest at physician appointments). Includes information about parents behavior change since MA-CORD.
	Increased linkages	Increase of collaboration, communication, and connections, either within the community or within the organization.
	Opportunities to implement new activities	Opportunities to implement or maintain new activities (eg, nutrition, physical activity, policies) with the help of MA-CORD.
	Opportunities to change policies, organizational environment, or both	Stakeholders talking about the opportunity to change policies, the organizational environment, or both to prevent and control childhood obesity with help of MA-CORD.
	Stakeholders’ behavior change, buy-in, and perceived responsibilities as role models	Change in stakeholders’ behaviors and how that might have influenced children’s behaviors.
	Stakeholders’ future intention to participate in MA-CORD	The answer to the interview question “If you were given the choice to be part of MA-CORD again, would you chose to?” was coded here.

**Lessons learned**	Leadership and administrative support	Information given about the importance of support needed to implement MA-CORD (eg, leadership, staff, administration).
	Preparation for unexpected changes	Any information about unforeseen events (eg, staff turnover, new hiring, weather) that were problematic during the implementation process.
	Early involvement of stakeholders to assess existing resources	All information on the importance to involve stakeholders early in the process (eg, for needs assessment).
	Regular communication	When stakeholders talked about lack of communication or the support of a good communication and communication tools (eg, within the MA-CORD team, within the program itself, within the sector).
	Account for family life circumstances and other barriers	Barriers and circumstances families face in preventing childhood obesity.

Abbreviation: MA-CORD, Massachusetts Childhood Obesity Research Demonstration.

## Results

Of the 40 stakeholders, 20 were from schools, 8 from health care, 4 from after-school programs, 3 from WIC, 2 from parks and recreation, and 3 were coordinators from the communities (Table 2). A summary of key successes and lessons learned follows, along with an illustrative quote. Additional quotes are provided in Table 3.

**Table 2:** Demographic Characteristics of Community Stakeholders (N = 40) in Study on Success Stories and Lessons Learned by Stakeholders in the MA-CORD Project, Massachusetts, 2013–2014

Characteristic	All, N = 40	Community 1, n = 19	Community 2, n = 21
**Community sector**			
School	20	10	10
Health care	8	4	4
After-school programs	4	1	3
Special Supplemental Nutrition Program for Women, Infants, and Children	3	2	1
Community coordinators	3	1	2
Parks and recreation department	2	1	1
**Sex**			
Female	36	16	20
Male	4	3	1
**Age, y**			
18–29	2	0	2
30–39	7	5	2
40–49	8	4	4
50–59	17	7	10
≥60	6	3	3
**Ethnicity**			
Not Hispanic	38	18	20
Hispanic	2	1	1
**Race**			
White	36	17	19
Asian	1	0	1
African American	1	1	0
Unknown	2	1	1

Abbreviation: MA-CORD, Massachusetts Childhood Obesity Research Demonstration.

**Table 3:** Main Themes, Subthemes, and Illustrating Quotes in Study on Success Stories and Lessons Learned by Stakeholders (N = 40) in the MA-CORD Project, Massachusetts, 2013–2014

Main Theme/Subtheme	Quote
**Success stories**	
Intervention acceptability	“Oh, it’s a high priority because it just kind of goes along with what we’re trying to do.” (WIC)
	“It is right up there with my priorities, because if we don’t have healthy kids, we aren’t gonna have kids in school to educate.” (School)
	“Some of the wellness policies for the city are now going back into the school and then into individual schools. I think it’s all tied in well, and right around the same time. MA-CORD, I think, helped to strengthen that message.” (School)
Increase in parent involvement	“I think there’s certainly in our community just a heightened awareness because of all the efforts that have been done to raise awareness around youth obesity. I certainly think because of the work in all the sectors that there’s awareness.” (Community coordinator)
Increased linkages	“Some other successes, our peer leaders are . . . going to the Healthy Weight Clinic. They’re gonna start going there once a month to help just do activities for kids and promote the five healthy behaviors for the kids going to the Healthy Weight Clinic.” (Parks and recreation)
Opportunities to implement new activities	“A couple of the things that we were working on was limiting screen time, serving 100 percent water outside of snack ’cause we serve milk with snack, and to ensure that all children get vigorous physical activity at least 15 minutes a day.” (After-school program)
Opportunities to change policies, organizational environment, or both	“There’s been a lot of policy changes, I guess you could say, in looking very closely at improving activity opportunities and nutritional value and nutritional — what can be eaten in school and what shouldn’t be.” (School)
	“I mean, we have no more vending machines. We have water easily accessible to everybody in the health center, including patients, staff.” (Health care)
Stakeholders’ behavior change, buy-in, and perceived responsibilities as role models	“We mirror what we’re trying to teach them. I’m trying very hard to work on the workplace wellness to emulate all of those messages for kids so that it is a constant stream of information and they’re not getting mixed messages.” (Health care)
	“Because I think I have to model it. If I don’t value it, no one else is gonna value it. People look to the leadership to see what’s of a value to them. If they look at the leadership and realize it is not of value to the leadership, they won’t get behind it.” (School)
	“When I first changed the policies for the staff handbook, there was no negative feedback. They completely understood, and they understood that they have to be the positive role models.” (After-school program)
	“We can’t just preach it to the kids, we have to model it.” (After-school program)
Stakeholders’ future intention to participate in MA-CORD	“’Cause I think it’s so important. I think that we need to focus on these things. WIC is a perfect partner to help with that because of the number of kids that we see, the number of families that we interact with and have a positive effect on them. Absolutely, I would hate to see us not participate.” (WIC)
	“I think that the concept and the structure of it is a really good model for other communities to follow. I feel like policy system and environmental change really provide the biggest impact at the community level, versus working with individual-level behavior change. Then, I feel like the model, in terms of all the sectors, with the consistent messaging, is also [a] best practice that other communities should be looking into. Everyone is on the same page with a common vision.” (Parks and recreation)
	”I would. I think it’s a good program.” (School)

**Lessons learned**	
Leadership and administrative support	“It matters to the superintendent. It matters to the mayor. It matters obviously to the school committee as well, but it matters to our PTO [parent–teacher organization], because the PTO has said to me that it’s not as vibrant at other schools because they feel that the principal is not pushing it as much as I am.” (School)
	“My director and manager are super supportive and continuously praising us.” (Health care)
Preparation for unexpected changes	“Because of the budget cuts and people’s positions being lost, there was a lot of movement this month. We have some folks that are teaching fourth and fifth grade this year, who were not teaching at that grade level last year, so we have new people to train.” (Community coordinator)
	“We’ve had to do more with less staff due to budget cuts.” (WIC)
	“Then we also have some brand new staff that are really new to [MA-CORD]. They don't know the bigger picture . . . and that’s a little more time-consuming getting them up to speed.” (School)
Early involvement of stakeholders to assess existing resources	“I have one school that was like, ‘Oh my! This is perfect! We needed it so much!’ Then I have another school . . . [the physical activity equipment] sat in boxes in the nurse’s office for three months.” (School)
	“Some of the things that were being discussed on the conference call, as a team, we had already established here or we already had those types of things in place here.” (Health care)
Regular communication	“I like listening to different ideas as other schools have done things, so if they have a forum or a blog that we could share information. I think that would be really helpful, because . . . if other schools that have the same kind of demographics that we have, if they’ve tried something that works, and vice versa, it would be great to hear, so we’re not trying to reinvent the wheel. It would take less time and energy to get something in place if they, if some school’s already done it.” (School)
	“And again it’s an opportunity to share information and share ideas and help each other. That’s been really helpful.” (After-school program)
Account for family life circumstances and other barriers	“Like I was telling you earlier, our participants are coming in with a range of needs, including housing, lack of food, other social issues. Sometimes nutrition is not what we talk about.” (WIC)

Abbreviations: MA-CORD, Massachusetts Childhood Obesity Research Demonstration; WIC, Special Supplemental Nutrition Program for Women, Infants, and Children.

### Success stories

**Intervention acceptability.** Most stakeholders (24 of 39, 62%) supported the program and made it a medium or high priority, and most (27 of 39, 69%) felt that MA-CORD fit into their organization, for example, by delivering similar messages. One stakeholder from WIC said, “I think [MA-CORD] should just be a normal part of everyone’s curriculum and messaging.”

**Increase in parent involvement.** About half of stakeholders (20 of 39, 51%) reported an increase in parent involvement. They observed higher participation rates in activities at schools and after-school programs, increased involvement during appointments at health care and WIC offices, and children bringing more healthful lunches to school. Stakeholders pointed to consistent messaging about 5 key behaviors throughout the community, an increase in community-wide strategies, and awareness of childhood obesity as reasons for these changes. A health care stakeholder noted, “The parents are asking questions. They’re more engaged when they come in for the visit. . . . Parents are actually coming over to the table asking questions, asking for the brochures — never happened before.”

**Increased linkages.** Two-thirds of stakeholders (26 of 39; 67%) reported improved connections to community resources, such as food services, the Safe Routes to School program, Head Start, and several community parks. Nine (23%) stakeholders said that visible and consistent messaging about MA-CORD and events helped to create linkages between community agencies and foster greater collaboration within organizations. As a WIC stakeholder noted, “We counsel on these same messages, so it’s great that they’re hearing it out in the community, too, whether it be at Head Start, at the park, at different after school programs.”

**Opportunities to implement new activities.** Most stakeholders (35 of 39; 90%) participating in MA-CORD were able to implement new activities to support increased physical activity and improved nutrition, such as regular walks to school, providing physical activity equipment, adding more healthful choices for breakfast and lunch in schools, offering more fruits and vegetables in schools and after-school programs, and changing menu options in public restaurants. One school stakeholder mentioned, “I’ve always done something with a walking program, but I really focused a lot on that. We have a walking club. I do it every morning early on. A lot of these things have started or have continued because of the program.”

**Opportunities to change policies and/or organizational environment.** About half of stakeholders (20 of 39; 51%) talked about changes in the policy or food environments, such as eliminating vending and soda machines, providing water instead of soda, and changing the staff handbook to discourage staff consumption of unhealthful snacks in front of the children. A school stakeholder noted the following:

[The school] took the chocolate milk right off the menu. The kids have white milk or water. . . . The girl that I work with, she said . . . ‘The white milk tastes like plastic.’ Then after a while she says, “Now that I had the white milk . . . I’m getting used to the taste. I had the chocolate milk and it’s so sweet.

**Stakeholders’ behavior change, buy-in, and perceived responsibilities as role models.** Sixteen (41%) stakeholders reported positive changes in staff and child behaviors. In schools, several stakeholders reported that school staff made more healthful choices to model behaviors and that children subsequently changed their eating behaviors. As one teacher said, “I used to bring in a salad every morning. . . . My students actually started doing the same. Instead of eating chips and cupcakes and cookies every day, I’d say probably at least one-third of my kids started bringing in salads in the morning and healthy snacks.”

Nine (23%) stakeholders indicated that awareness about childhood obesity and the 5 key behaviors increased. Stakeholders mentioned that they are more aware than before that children are watching too much television or eating too much sugar or that parents are sending requests for more information about the MA-CORD program.

All stakeholders said that they would participate in MA-CORD again, because they were aware of the childhood obesity problem and the impact it was having on their communities and because they believed in the program, as stated from a stakeholder from a parks and recreation department.

I think that the concept and the structure of it [MA-CORD] is a really good model for other communities to follow. I feel like policy, system, and environmental change really provides the biggest impact at the community level, versus working with individual-level behavior change. Then . . . in terms of all the sectors, with the consistent messaging, is also best practice that other communities should be looking into. Everyone is on the same page with a common vision.

### Lessons learned

**Leadership and administrative support.** Almost all stakeholders (35 of 39; 90%) reported that the presence of leadership and administrative support for the program reduced feelings of conflict between program implementation and other priorities among staff members. A school stakeholder mentioned, “We have very, very good support . . . with the principals in each building. They’re extremely approachable about anything that we ask. If we say, ‘Hey, you’ve got an assembly coming up. . . . Can one of those have a MA-CORD component?’ They’re like, ‘Okay.’”

Likewise, the challenges resulting from a lack of buy-in from leaders were described by a school stakeholder who experienced challenges with program implementation when administrative support waned: “They do not even mention it [MA-CORD] anymore. . . . Last year it was ‘We want you to do this curriculum,’ and this year it’s not even mentioned by the administration.”

**Preparation for unexpected changes.** Most stakeholders (22 of 39; 56%) named several unforeseen events during planning and implementing MA-CORD. Turnover caused by retirements, job loss, and resignations was experienced at all levels of staff. A stakeholder from the health care sector said, “The school department, they’re so understaffed right now. . . . Trying to get into the school department to try to spread the message or be involved is tough.”

Also, new staff were hired and became part of the implementation process. Another unpredictable event was inclement weather, which lead to cancellations of many trainings in the school and after-school sectors, causing delays in program implementation.

**Early involvement of stakeholders to assess existing resources.** Twelve stakeholders mentioned the importance of assessing the processes and tools that organizations have in place before planning and implementing interventions. They mentioned that they already had access to resources (eg, a system to track height and weight in the clinical sector) and informational material on childhood obesity prevention before MA-CORD was implemented, and either did not understand why their systems should change, or did not find the changes helpful. A stakeholder from the healthcare sector noted, “A lot of the things that they’re discussing now, we’ve already learned or done.”

**Regular communication.** More than half of the stakeholders (23 of 39; 59%) wished for more regular communication and greater clarity about their role in MA-CORD, as described by a school stakeholder:

I’ll be honest with you, I wish I knew more of what was available through MA-CORD. . . . There were a couple of your colleagues here . . . and they were telling me all the things that were available, and I was like, “I didn’t know any of that.” . . . Sometimes communication in the district is a little difficult. I just wish I knew more about what was available to us.

Cross-sector communication was particularly important. Twelve (31%) stakeholders cited the benefits of exchanging information and ideas during cross-sector training sessions, which helped them to explore new ideas and to discuss their experiences with intervention components and events they had planned. Additionally, stakeholders addressed a communication tool, such as an online platform as opportunity to discuss what is and is not working. An afterschool stakeholder said, “The opportunity to share with the other teams and hear what they’re doing, working with the administrators of the program and the specialists to get ideas has been good.”

**Account for family life circumstances and other barriers.** Although a range of strategies were used to accommodate the various needs of families to improve involvement in MA-CORD, 19 (49%) stakeholders named families’ lack of financial support and transportation challenges as two of the most common reasons for low program attendance. One WIC stakeholder mentioned, “Our participants are coming in with a range of needs including housing, lack of food, other social issues. Sometimes nutrition is not what we talk about.”

## Discussion

Overall, we found a high level of stakeholder and community buy-in to MA-CORD with all stakeholders reporting they would implement MA-CORD again. Stakeholders said that the program was a priority for their organization because it was consistent with their organization’s goals and provided opportunities to implement new and old activities and policies and support existing ones. Other studies show that changing existing policies or using new policies can ensure program sustainability (19). A novel finding of this study is that stakeholders served as positive role models for families and were motivated to change their own behaviors. These successes may be due to the fact that MA-CORD was implemented by community organizations rather than by researchers. This type of experiential learning can be a motivational tool for behavior change when working with community stakeholders.

Half of all stakeholders described increases in parent participation in activities. Parent involvement is necessary for successful implementation of child health interventions (13,25,26). MA-CORD used diverse strategies for approaching and involving parents; these strategies ranged from in-person counseling at WIC and health care visits, school events that included a MA-CORD media competition (27), and materials promoting the 5 target behaviors that were distributed across sectors. Stakeholders also observed that families faced many challenges beyond nutrition; these are described elsewhere (28). In future interventions, parent involvement could be further enhanced through a more holistic approach that moves beyond a focus on children’s diet and physical activity.

Although levels of community and stakeholder buy-in were high in both communities, levels of administrative and leadership support were sometimes low. During these periods, other events, such as an anti-bullying program, were given higher priority. A strong communication strategy directed toward administrators and leaders can help gain their necessary support. Regular staff turnover, particularly in schools, created challenges, because training new staff was logistically problematic. Developing a comprehensive training manual and using a train-the-trainer model may have alleviated some of these challenges. Unforeseen events can be addressed effectively if the project anticipates these possibilities from the beginning. Training sessions were often difficult to reschedule given the number of people involved. In the future, it may be advisable to prepare web-based trainings as alternative. Finally, stakeholders were enthusiastic about cross-sector interactions and communication. However, few of these opportunities were provided in MA-CORD. Future programs would benefit from creating multiple opportunities for cross-sector training and learning collaborations to permit the sharing of resources and lessons learned.

Qualitative studies add to existing epidemiological and behavioral evidence because they may suggest ideas for adapting interventions to community and individual needs (29). This study has several limitations. First, a low response rate could indicate a selection effect in which only the stakeholders most committed to MA-CORD chose to participate. Another limitation was the use of convenience sampling. Aside from stakeholders’ existing involvement with MA-CORD, no other exclusion criteria were defined. As a result, our sample over-represents stakeholders from the school sector. Because we invited all eligible stakeholders to participate, chances were high that a higher portion of school participants would be interested in participating. Finally, because MA-CORD was implemented only in 2 low-income communities in the northeastern United States, findings may not be generalizable to all communities; however, providing a detailed description about the study sample and the 2 intervention communities may still help other researchers to apply our results to their studies (17).

This study contributes to implementation research by identifying important successes and lessons learned in the context of a multisite and multisector program to prevent and control childhood obesity. The insight gained through this process will benefit future interventions by streamlining the implementation processes and anticipating challenges before they occur (18).

## References

[1] Ogden CL , Carroll MD , Kit BK , Flegal KM . Prevalence of childhood and adult obesity in the United States, 2011–2012. JAMA 2014;311(8):806–14. 10.1001/jama.2014.732 24570244 PMC4770258

[2] Morgenstern M , Sargent JD , Hanewinkel R . Relation between socioeconomic status and body mass index: evidence of an indirect path via television use. Arch Pediatr Adolesc Med 2009;163(8):731–8. 10.1001/archpediatrics.2009.78 19652105 PMC3719170

[3] Shrewsbury V , Wardle J . Socioeconomic status and adiposity in childhood: a systematic review of cross-sectional studies 1990–2005. Obesity (Silver Spring) 2008;16(2):275–84. 10.1038/oby.2007.35 18239633

[4] Jones-Smith JC , Dieckmann MG , Gottlieb L , Chow J , Fernald LC . Socioeconomic status and trajectory of overweight from birth to mid-childhood: the Early Childhood Longitudinal Study-Birth Cohort. PLoS One 2014;9(6):e100181. 10.1371/journal.pone.0100181 24950056 PMC4065031

[5] Gortmaker SL , Swinburn BA , Levy D , Carter R , Mabry PL , Finegood DT , Changing the future of obesity: science, policy, and action. Lancet 2011;378(9793):838–47. 10.1016/S0140-6736(11)60815-5 21872752 PMC3417037

[6] Davison KK , Birch LL . Childhood overweight: a contextual model and recommendations for future research. Obes Rev 2001;2(3):159–71. 10.1046/j.1467-789x.2001.00036.x 12120101 PMC2530932

[7] Institute of Medicine. Accelerating progress in obesity prevention: solving the weight of the nation. Washington (DC): Institute of Medicine; 2012.

[8] Addy NA , Shaban-Nejad A , Buckeridge DL , Dubé L . An innovative approach to addressing childhood obesity: a knowledge-based infrastructure for supporting multi-stakeholder partnership decision-making in Quebec, Canada. Int J Environ Res Public Health 2015;12(2):1314–33. 10.3390/ijerph120201314 25625409 PMC4344668

[9] World Health Organization. Population-based approaches to childhood obesity prevention. 2012. http://www.who.int/dietphysicalactivity/childhood/WHO_new_childhoodobesity_PREVENTI N_27nov_HR_PRINT_OK.pdf. Accessed October 17, 2016.

[10] Ewart-Pierce E , Mejía Ruiz MJ , Gittelsohn J . “Whole-of-community” obesity prevention: a review of challenges and opportunities in multilevel, multicomponent interventions. Curr Obes Rep 2016;5(3):361–74. 10.1007/s13679-016-0226-7 27379620 PMC5962013

[11] Creswell JW . Qualitative inquiry and research design — choosing among five approaches. 2nd edition. Los Angeles (CA): SAGE Publications; 2007 .

[12] World Health Organization. Prioritizing areas for action in the field of population based prevention of Childhood Obesity. A set of tools for member states to determine and identify priority areas for action. 2012. http://www.who.int/dietphysicalactivity/childhood/Childhood_obesity_Tool.pdf

[13] Golan M , Crow S . Parents are key players in the prevention and treatment of weight-related problems. Nutr Rev 2004;62(1):39–50. 10.1111/j.1753-4887.2004.tb00005.x 14995056

[14] Centers for Disease Control and Prevention. The social-ecological model: a framework for prevention. 2015. https://www.cdc.gov/violenceprevention/overview/social-ecologicalmodel.html. Accessed December 18, 2016.

[15] Dooyema CA , Belay B , Foltz JL , Williams N , Blanck HM . The childhood obesity research demonstration project: a comprehensive community approach to reduce childhood obesity. Child Obes 2013;9(5):454–9. 10.1089/chi.2013.0060 24094146

[16] Davison KK , Falbe J , Taveras EM , Gortmaker S , Kulldorff M , Perkins M , ; MA-CORD Study Group. Evaluation overview for the Massachusetts Childhood Obesity Research Demonstration (MA-CORD) project. Child Obes 2015;11(1):23–36. 10.1089/chi.2014.0059 25575095 PMC5915219

[17] Taveras EM , Blaine RE , Davison KK , Gortmaker S , Anand S , Falbe J , ; MA-CORD Study Group. Design of the Massachusetts Childhood Obesity Research Demonstration (MA-CORD) study. Child Obes 2015;11(1):11–22. 10.1089/chi.2014.0031 25469676 PMC4322791

[18] Oude Luttikhuis H , Baur L , Jansen H , Shrewsbury VA , O’Malley C , Stolk RP , Interventions for treating obesity in children. Cochrane Database Syst Rev 2009;3(1):CD001872. 19160202 10.1002/14651858.CD001872.pub2

[19] World Health Organization. Global strategy on diet, physical activity and health. 2014. http://www.who.int/dietphysicalactivity/childhood_why/en/. Accessed October 17, 2016.

[20] US Census Bureau. QuickFacts. 2015. http://www.census.gov/quickfacts/table/PST045215/2523875. Accessed October 17, 2016.

[21] US Census Bureau. QuickFacts. 2015. http://www.census.gov/quickfacts/table/PST045215/2545000. Accessed October 17, 2016.

[22] Foltz JL , Belay B , Dooyema CA , Williams N , Blanck HM . Childhood Obesity Research Demonstration (CORD): the cross-site overview and opportunities for interventions addressing obesity community-wide. Child Obes 2015;11(1):4–10. 10.1089/chi.2014.0159 25679059 PMC4322789

[23] Braun V , Clarke V . Using thematic analysis in psychology. Qualitative Research in Psychology 2006;3(2):77–101.

[24] Merriam SB . Assessing and evaluating qualitative research. In: Qualitative research in practice: examples for discussion and analysis. S. Merriam, editor. San Francisco (CA): Jossey-Bass; 2002. p. 22–29.

[25] Lindsay AC , Sussner KM , Kim J , Gortmaker S . The role of parents in preventing childhood obesity. Future Child 2006;16(1):169–86. 10.1353/foc.2006.0006 16532663

[26] Rhee KE , De Lago CW , Arscott-Mills T , Mehta SD , Davis RK . Factors associated with parental readiness to make changes for overweight children. Pediatrics 2005;116(1):e94–101. 15995022 10.1542/peds.2004-2479

[27] Criss S , Cheung L , Giles C , Gortmaker S , Viswanath K , Kwass JA , Media competition implementation for the Massachusetts Childhood Obesity Research Demonstration Study (MA-CORD): adoption and reach. Int J Environ Res Public Health 2016;13(4):403. 10.3390/ijerph13040403 27058549 PMC4847065

[28] Ganter C , Chuang E , Aftosmes-Tobio A , Blaine RE , Giannetti M , Land T , Community stakeholders’ perceptions of barriers to childhood obesity prevention in low-income families, Massachusetts 2012–2013. Prev Chronic Dis 2015;12:E42. 10.5888/pcd12.140371 25811497 PMC4375987

[29] Corrrigan M , Cupples ME , Smith SM , Byrne M , Leathem CS , Clerkin P , The contribution of qualitative research in designing a complex intervention for secondary prevention of coronary heart disease in two different healthcare systems. BMC Health Serv Res 2006;6(1):90. 10.1186/1472-6963-6-90 16848896 PMC1543625

